# Arsenic health risks and interaction with salinity in coastal areas of Bangladesh

**DOI:** 10.3389/fpubh.2025.1610311

**Published:** 2025-08-05

**Authors:** Islam Md Tawhidul, Khatun Mst. Asma, Moinul Islam, Koji Kotani

**Affiliations:** ^1^School of Economics and Management, Kochi University of Technology, Kochi, Japan; ^2^Department of Economics, Pabna University of Science and Technology, Pabna, Bangladesh; ^3^Research Institute for Future Design, Kochi University of Technology, Kochi, Japan; ^4^Department of Agricultural and Applied Statistics, Bangladesh Agricultural University, Mymensingh, Bangladesh; ^5^Urban Institute, Kyushu University, Fukuoka, Japan; ^6^College of Business, Rikkyo University, Tokyo, Japan

**Keywords:** water-related diseases, cardiovascular diseases, arsenic, salinity, Bangladesh

## Abstract

**Background:**

Arsenic contamination poses significant health risks to inhabitants in coastal areas. However, little is known about the health risks associated with the interaction of arsenic with salinity. This study aims to examine how the morbidities from water-related diseases (WRD) and cardiovascular diseases (CVD) are associated with arsenic contamination under salinity with multiple water sources and uses as well as how such risks can be reduced. It is hypothesized that WRD and/or CVD morbidity rates worsen when severe arsenic contamination comes with salinity, and there exist effective countermeasures for the risk reduction by different channels.

**Methods:**

We collected data from 689 households using questionnaire surveys on diseases, water uses, safety measures and sociodemographic factors from arsenic areas under salinity in Bangladesh and employed logit and ordered probit regression models to analyze the incidence and intensity of the morbidity.

**Results:**

The results reveal that, first, households in high arsenic contaminated areas have higher morbidity rates of WRD and/or CVD than those in no arsenic contaminated areas under salinity. Second, the daily use of washing and cooking water (drinking water) increases (does not increase) the probability of WRD and CVD morbidities, respectively. Third, households using deep tubewells as a safety measure face greater WRD morbidity than those using rainwater.

**Conclusion:**

The results suggest that cooking and washing are the main channels for increasing the health risks and two countermeasures are recommended: (i) extensive year-round uses of rainwater and (ii) adoption of regularly tested water sources, such as groundwater, even for cooking and washing to reduce the risks for achieving Sustainable Development Goal 3.

## 1 Introduction

Global concerns about environmental health risks are rising as pollution and climate change intensify public health challenges, especially in developing countries (1, 2). Among these risks, water contamination has emerged as a critical public health concern, with widespread implications for human health and wellbeing (3–5). Coastal Bangladesh faces high risks due to arsenic contamination and salinity intrusion, both of which significantly affect water quality and public health (6–8). These environmental adversities contribute to an increasing prevalence of cardiovascular diseases (CVD) and water-related diseases (WRD), further worsening health burdens in these vulnerable communities (9–12). Given these concerns, this study investigated the associations between varying levels of arsenic contamination under salinity and morbidities from WRD and CVD, aiming to identify effective strategies to safeguard the wellbeing of coastal populations.

Arsenic contamination poses a significant public health threat, including cardiovascular diseases (CVD) and water-related diseases (WRD) (13–16). Chen et al. (9) and Das et al. (10) evaluated the associations between arsenic exposure in drinking water and CVD risks using a prospective cohort of 11,746 individuals in Bangladesh and medical tests of 210 subjects in India, respectively, and the results revealed a significantly increased risk. Wade et al. (11) conducted a hospital-based case-control study with 573 subjects in China, measuring the arsenic concentrations in drinking water and toenail samples of patients, finding that each 10 μg/L increase in arsenic raised CVD risks by 19%. Some studies have indicated that arsenic contamination is correlated with a high prevalence of skin diseases (17, 18). Tondel et al. (19) performed a cross-sectional study in Bangladesh, measuring arsenic levels in drinking water and examining 1481 subjects, suggesting a direct relationship between arsenic levels and the occurrence of skin lesions. Maharjan et al. (20) conducted a community-based study in Nepal, measuring arsenic levels in 146 tubewells and examining 1,343 subjects, reporting 6.9% prevalence of arsenic-induced skin manifestations. The impact of arsenic in drinking water is well-documented. However, its effects through other channels, such as cooking, remain understudied, especially in areas where different water sources are used for various household purposes (21, 22).

Salinity in coastal areas, intensified by climate change, poses significant health risks, particularly cardiovascular diseases (CVD) and water-related diseases (WRD) (7, 23–25). Khan et al. (26) linked salinity-contaminated drinking water to seasonal hypertension in pregnancy, using historical salinity and hospital data, finding a positive correlation, particularly during the dry season. Talukder et al. (27) examined the associations between drinking water salinity and blood pressure in 253 young adults in Bangladesh through a cross-sectional study, and reported that high salinity (>600 mg/L) is significantly associated with elevated blood pressure. Nahian et al. (5) analyzed the health impacts of drinking water salinity using a multilevel regression model with data from over 1,500 households, finding that salinity intrusion increases the risk of hypertension. Chakraborty et al. (28) demonstrated that high salinity increases hospital visits for CVD, diarrhea and abdominal pain in Bangladesh using a cross-sectional survey with 157 subjects. Asma and Kotani (12) investigated the impact of coastal salinity on WRD in Bangladesh through a questionnaire survey with 527 subjects. They reported that the occurrence of the diseases is highly probable in salinity-affected areas, and rainwater consumption reduces the risk. Towhid et al. (29) showed that coastal children experience more frequent diarrhea and intestinal inflammation due to drinking water salinity than non-coastal children do. These studies highlighted the positive relationship between salinity contamination and morbidity from CVD and WRD, mainly through drinking water.

The literature has separately demonstrated the adverse health effects of arsenic contamination and salinity on CVD and WRD, with a primary focus on drinking water (5, 9–11, 27). However, little is known about the combined health risks associated with arsenic and salinity, as the interaction effects are expected to be significant in some coastal regions of the world. This study examines how the morbidities from WRD and CVD are associated with arsenic contamination under salinity with multiple water sources and uses, and how such risks can be reduced. We hypothesize that WRD and/or CVD morbidity rates worsen when severe arsenic contamination comes with salinity, and there exist effective countermeasures for the risk reduction by different channels. To answer these questions and hypotheses, the coastal regions of Bangladesh are chosen as our study areas where arsenic contamination and salinity coexist and people in these regions are familiar with multiple water sources, including surface, ground and rain and uses in daily different activities, such as drinking, cooking and washing, in consideration to various risks, water quality and scarcity (30–33). In these areas, we conducted questionnaire surveys with 689 households, collecting and analyzing the data on diseases, water uses, safety measures and sociodemographic factors.

This study addresses several important and current concerns in environmental health research. First, it addresses a critical gap in the environmental health literature by investigating how arsenic contamination and salinity jointly influence disease risks, especially under multiple household water sources. Although existing studies have examined arsenic and salinity independently, their potential interaction across drinking, cooking and washing water remains insufficient. Second, the findings are essential for developing context-specific countermeasures in coastal regions such as Bangladesh, where communities rely on diverse water sources. Understanding these risk pathways is crucial for reducing the burden of WRD and CVD, enhancing public health outcomes, and guiding sustainable water use practices in line with the Sustainable Development Goal 3 (SDG 3). Thus, this research contributes to both academic knowledge and the practical implementation of health and water management strategies in vulnerable coastal areas.

## 2 Arsenic and salinity contamination in Bangladesh

Arsenic contamination in Bangladesh is one of the most severe environmental and public health crises, affecting millions of people (34–38). The contamination appeared in the 1990s when groundwater was extensively utilized for drinking and irrigation, predominantly in the Ganges delta region (35, 39, 40). Approximately 61 out of 64 districts are affected, with arsenic levels often exceeding both the national 50 μg/L and World Health Organization (WHO) standard 10 μg/L (41–43). An estimated 75–80 million people are at risks, with 24–30 million already exposed to toxic levels (36, 44). The contamination, which is largely geogenic, originates from geological deposits and is exacerbated by the widespread use of shallow tubewells installed in 1970s to provide safe drinking water (45). Chronic exposure has led to severe health impacts, including skin lesions, cancers, cardiovascular diseases, and neurological disorders, particularly among children and malnourished individuals (35, 37). Despite mitigation efforts such as deep tubewells and arsenic removal technologies, sustainable solutions remain challenging due to the scale of contamination and socioeconomic constraints (46).

Salinity contamination is a growing environmental and public health challenge in coastal Bangladesh, affecting soil, water resources, agriculture and human health (28, 47, 48). This problem is driven by climate change, rising sea levels, reduced upstream freshwater flow and human activities, such as shrimp farming and excessive groundwater extraction (47, 49). Coastal areas, which constitute 32% of the country's land, face increasing soil and water salinity due to saltwater intrusion (50). The affected area has expanded from 83.3 million hectares in 1973 to 105.6 million hectares in 2009 (49). Salinity levels fluctuate seasonally, intensifying during the dry season as saltwater extends inland by 240 km, contaminating both surface water and groundwater (51, 52). This poses severe risks to freshwater resources, making drinking water unsafe and increasing salt intake in coastal communities, where the estimated intake reaches 5–16 g/day during the dry season, far exceeding the recommended limits (26). The health risks of salinity exposure include hypertension, cardiovascular diseases and pregnancy complications (53). Adaptive strategies such as rainwater harvesting and filtering have been implemented. However, finding sustainable solutions remains challenging due to the scale of the problem (48).

## 3 Methodology

**Figure 1:**
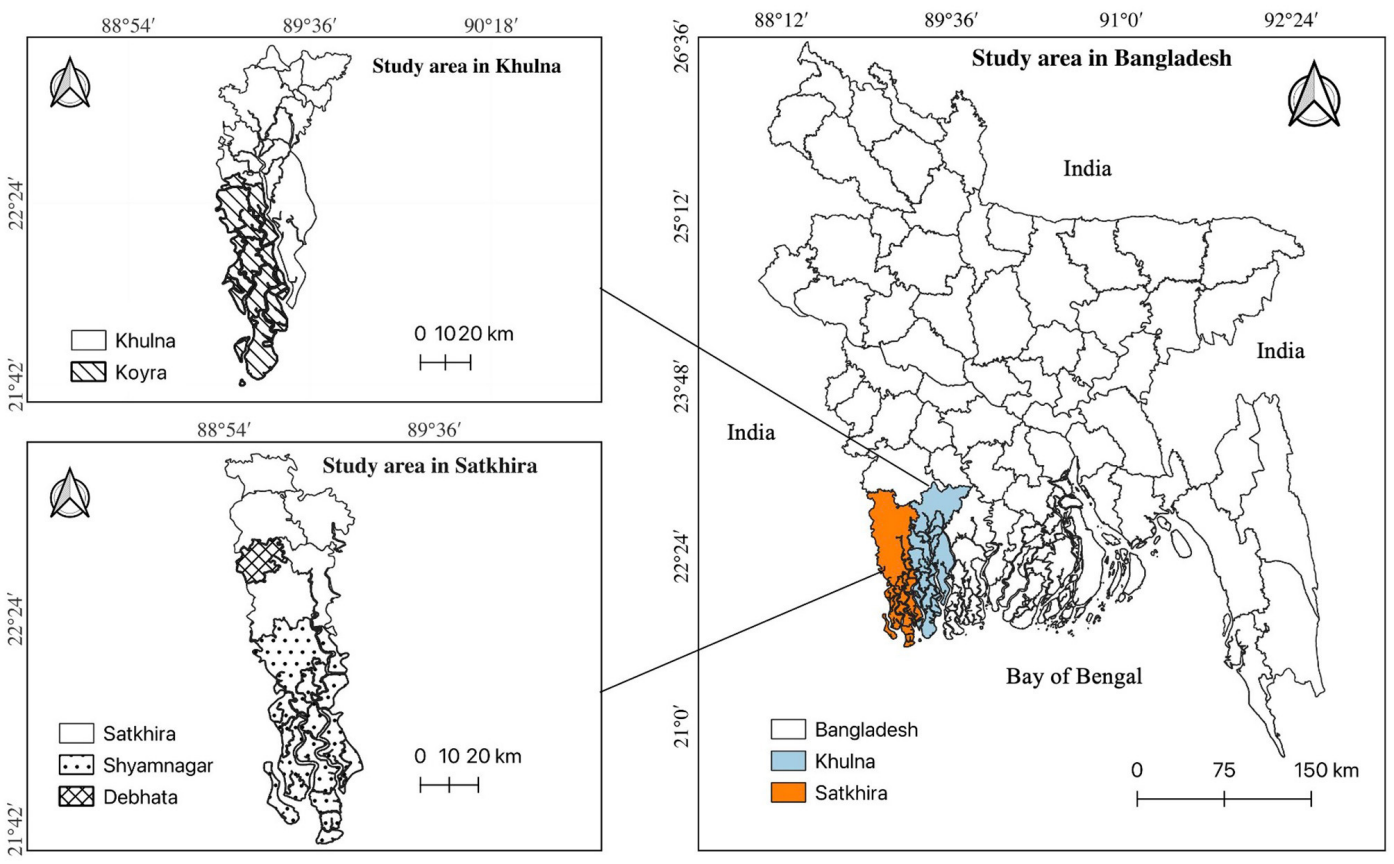
Location of study areas in Bangladesh.

Field surveys were conducted in the coastal districts of Satkhira and Khulna in southwestern Bangladesh (Figure 1). Two subdistricts from Satkhira, namely, Shyamnagar and Debhata, and one subdistrict from Khulna, namely, Koyra, were selected for this study. These areas face significant salinity intrusion, primarily due to rising sea levels and frequent flooding (47, 54). The average salinity in these areas is ~2,000 parts per million (ppm) in surface water and ~1,000 ppm in groundwater (6, 55). Additionally, these areas are affected by varying levels of arsenic contamination, with Shyamnagar having on average less than 10 parts per billion (ppb) (< 10 ppb), Debhata between 10 and 50 ppb (10 ≤ ppb ≤ 50) and Koyra exceeding 50 ppb (>50 ppb) (6, 56, 57). According to World Health Organization (WHO) standards, arsenic levels below 10 ppb are acceptable for drinking water. Based on the arsenic levels and the arsenic risks categorization proposed by the WHO, Shamsudduha et al. (8) and Charles et al. (58), we classified Shyamnagar as no, Debhata as medium and Koyra as high arsenic areas under similar salinity level. To confirm the arsenic levels during the field survey, arsenic in drinking, cooking and washing water was randomly measured from 10 households in each subdistrict with the assistance of local non-governmental organizations (NGOs). The measured arsenic concentrations closely matched with previously reported levels (see Table A1 in the Appendix).^1^

**Figure 2:**
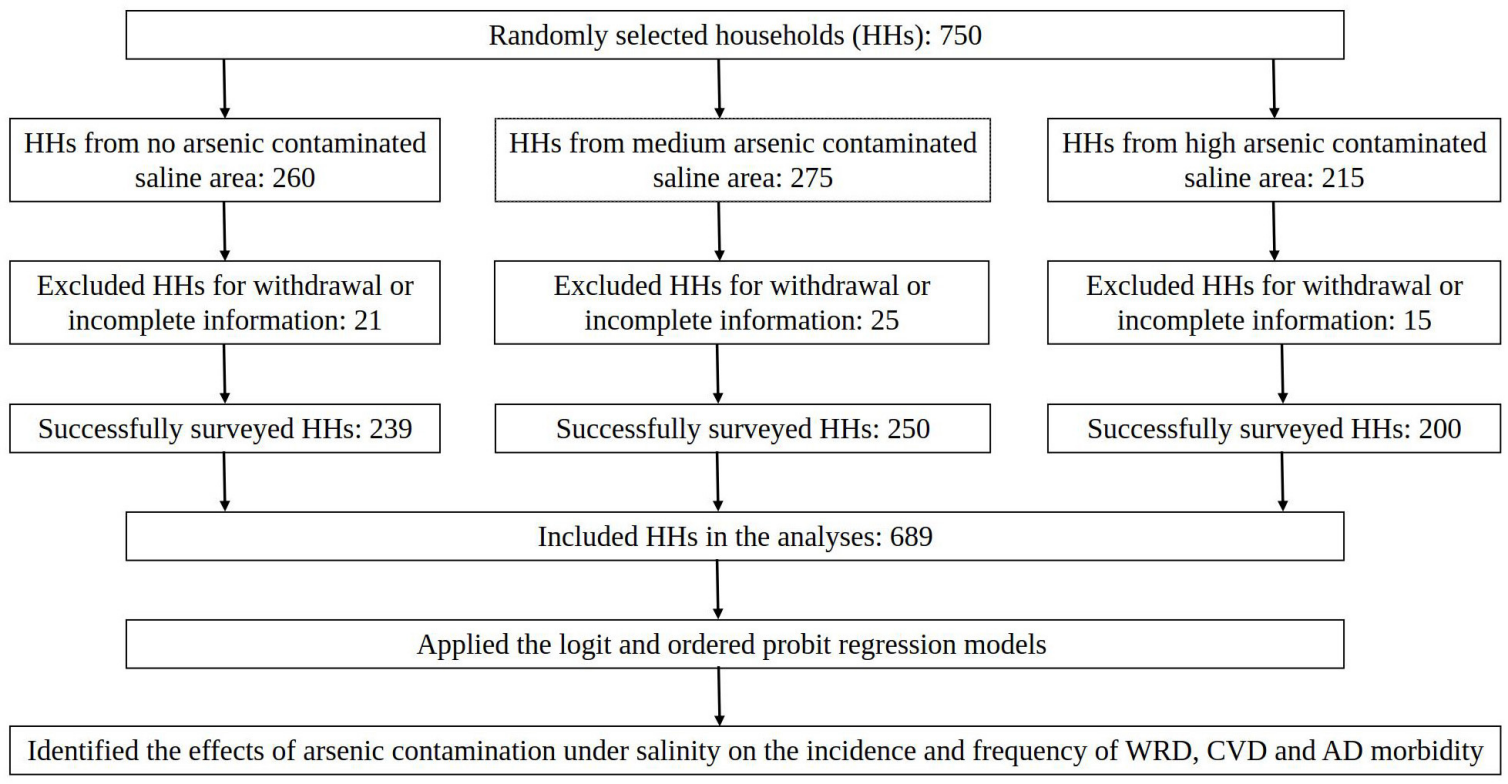
A methodological flowchart of the study.

In this study, a random sampling procedure was implemented to select six villages from each subdistrict. Local NGOs assisted in obtaining lists of all households within these villages. From these lists, 750 households were chosen using a random number generator, with 260 selected from Shyamnagar, 275 from Debhata and 215 from Koyra, proportional to the total population of each subdistrict. A total of 689 households were successfully surveyed, including 239 from Shyamnagar, 250 from Debhata and 200 from Koyra, whereas 61 observations were excluded due to respondent withdrawal or incomplete information. A methodological flowchart illustrating the sampling procedure for this study is shown in Figure 2. The second author served as the chief administrator of the survey and provided training to the research assistants on data collection and survey conduct. Trained research staff conducted the surveys using a predefined questionnaire. The household head (husband or wife) participated willingly, signing a written consent form at the beginning of the survey.

Table 1 presents the names and descriptions of the dependent and independent variables. The dependent variables include water-related diseases (WRD), cardiovascular diseases (CVD) and any diseases (AD) morbidity in the study areas. This study focuses on five common WRD, including respiratory problems, diarrhea, skin diseases, allergic rhinitis and reduced lung function, and four CVD, including high blood pressure, heart problems, stroke and breath shortening. Following the steps proposed by Asma and Kotani (12), the survey first inquired about any morbidity experienced by the household head in the last six months. If no morbidity was reported, the questions were extended to other family members. Data were recorded for one family member per household who had suffered from diseases, with priority given to adult members if multiple were affected. Supporting evidence of diseases, such as prescriptions or diagnostic reports, was also collected, particularly when participants had difficulty recalling details. The analysis extends to any diseases (AD), referring to the occurrence of either WRD or CVD. The study also measures the intensity of morbidity through the frequency of water-related diseases (FWRD), cardiovascular diseases (FCVD) and any diseases (FAD), capturing the number of simultaneous diseases experienced by a household member.

**Table 1:** Definitions of variables.

**Variables**	**Descriptions**
**Dependent variables**	
Water-related diseases (WRD)	If a household member experiences morbidity from any water-related disease, then the value is 1, otherwise 0.
Cardiovascular diseases (CVD)	If a household member experiences morbidity from any cardiovascular disease, then the value is 1, otherwise 0.
Any diseases (AD)	If a household member experiences morbidity from any type of WRD or CVD, then the value is 1, otherwise 0.
Frequency of water-related diseases (FWRD)	The frequency of a household member suffering from one or more water-related diseases.
Frequency of cardiovascular diseases (FCVD)	The frequency of a household member suffering from one or more cardiovascular diseases.
Frequency of any diseases (FAD)	The frequency of a household member suffering from one or more any diseases (WRD or CVD).
**Independent variables**	
Arsenic areas under salinity (Base group = No arsenic)	
Medium	If a household is in a medium arsenic contaminated saline area, then the value is 1, otherwise 0.
High	If a household is in a high arsenic contaminated saline area, then the value is 1, otherwise 0.
Water uses	
Drinking	Liter (L) per day per household
Cooking	Liter (L) per day per household
Washing	0–100 L per day per household is 1, otherwise 0.
Water safety measures (Base group = rainwater)	
Deep tubewell	If a household takes deep tubewell as a water safety measure, then the value is 1, otherwise 0.
Other	If a household takes other or no water safety measure, then the value is 1, otherwise 0.
**Sociodemographic variables**	
Age	Expressed by years
Gender	1 when a subject is female, otherwise 0.
Occupation of household head	1 when occupation is agriculture, otherwise 0.
Education of household head	Years of schooling (0 to 14) (0 = No schooling, 1 to 12 = class one to twelve, 13 = Graduate/equivalent, 14 = Post graduate/equivalent )
Family structure	1 when family is nuclear, otherwise 0.
Household income	Monthly household income in BDT^*a*^

^a^BDT stands for Bangladeshi currency “Taka.”

The independent variables include no, medium and high arsenic areas under salinity, each coded as 1 for households located in these areas and 0 otherwise. Water use variables focus on the daily consumption of drinking, cooking and washing water per household. Daily drinking and cooking water is measured in liters, whereas washing water is categorized as 1 if a household uses 0-100 L and 0 if more than 100 L/day.^2^ Water safety measures is coded as 1 for households using deep tubewells as a safety measure and 0 otherwise. Sociodemographic data include age, gender, occupation, education of the household head, family structure and household income. This study evaluates the impact of these independent variables on household disease morbidity.

The average, median and standard deviation are computed for each of the dependent and independent variables. Pie charts are then used to illustrate the percentages of WRD, CVD and AD morbidities across contaminated areas. We employ logit regression models to analyze the effects of independent variables on the probability of a household suffering from WRD, CVD or AD. In these models, $Y_{i}^{K}s$ represent the dependent variables where *K* denotes any of the WRD, CVD and AD morbidities. Here, $Y_{i}^{K}=1$ if a member of a household *i* has suffered from a disease, and $Y_{i}^{K}=0$ otherwise. The probability of a household suffering from WRD, CVD or AD, denoted by $\mathrm{Pr}\left(\overset{K}{\underset{i}{Y}}=1\right)$, is assumed to follow the *F* distribution function evaluated at ${X_{i}\beta }^{K}$. Here, ***X*****_*i*_** is a 1 × (*m*+1) vector of explanatory variables for household *i* (***X*****_*i*_** = (1, *X*_*i*1_, …, *X*_*im*_)) and **β**^*K*^ is a (*m*+1) × 1 vector of parameters $\left(\beta^{K}={\left(\beta_{0}^{K},\beta_{1}^{K},\ldots ,\beta_{m}^{K}\right)}^{\prime }\right)$. The distribution function of the logit regression model is as follows:


(1) $$\begin{array}{l} \mathrm{Pr}\left(Y_{i}^{K}=1\right)=\frac{\exp\left({X_{i}\beta }^{K}\right)}{1+\exp\left({X_{i}\beta }^{K}\right)}. \end{array}$$

A specification of Equation 1 enables us to compute **β**^*K*^ via maximum likelihood methods to characterize $Y_{i}^{K}$ (59–61). To ensure the robustness of our findings, a series of logit regression models are applied sequentially. Initially, we examine the relationship between disease occurrence and arsenic areas under salinity. We subsequently incorporate variables related to water uses and water safety measures. Finally, sociodemographic variables are added to the models. The similar quantitative results are consistently observed across all the models, underscoring the robustness of our findings. The principal findings from these logit regression analyses are summarized in Table 4.

The ordered probit model is utilized in this study to estimate relationships between an ordinal dependent variable and the independent variables (59, 62). This approach is particularly beneficial when the dependent variable represents sequential outcomes, making it ideal for evaluating the frequency of events, such as disease morbidity, in the study areas. The ordered probit model is formulated as follows:


(2) $$\begin{array}{l} Y_{i}^{*K}=\gamma_{0}^{K}+{X_{i}\gamma }^{K}+\varepsilon_{i}^{K} \end{array}$$

where $Y_{i}^{*K}$ is an unobserved dependent variables measuring the frequency of disease morbidity by a household member *i*. *K* denotes the FWRD, FCVD and FAD, with frequencies ranging from 1 to 5 for FWRD, 1 to 4 for FCVD and 1 to 9 for FAD. ***X*****_*i*_** is the vector of the explanatory variables, such as areas, water uses, water safety measures and sociodemographic variables. γ_0_ is the parameter associated with the intercept, **γ**^*K*^ = (γ_1_, γ_1_, …, γ_13_) is the vector of unknown parameters associated with ***X*****_*i*_** and $\varepsilon_{i}^{K}$ is a random error term assumed to be normally distributed with zero mean and unit variance. The main results of the ordered probit models are summarized in Tables 5, 6. All the statistical analyses were conducted using Stata 17 (StataCorp LLC, College Station, TX, USA).

## 4 Results

**Figure 3:**
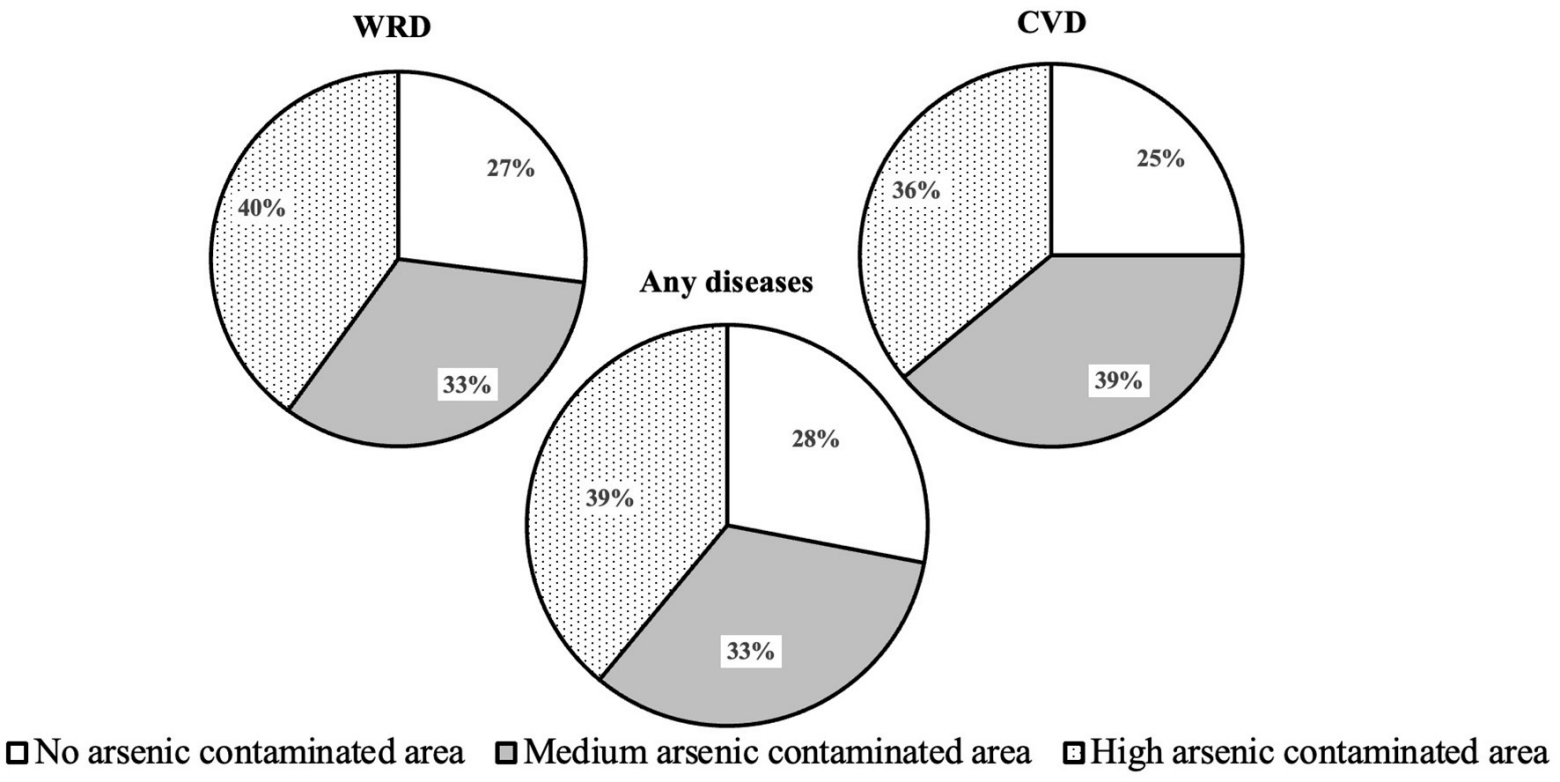
The percentages of water-related diseases (WRD), cardiovascular diseases (CVD) and any diseases morbidity by contaminated areas.

Table 2 presents the average, median and standard deviation of the dependent variables, such as water-related diseases (WRD), cardiovascular diseases (CVD) and any diseases (AD) (any of WRD or CVD) morbidities across varying arsenic contamination levels under similar salinity level. The percentages of households experiencing WRD (CVD) morbidity in no, medium and high arsenic areas under salinity are 34 (16), 39 (22) and 48% (21%), while AD morbidity rates are 41, 48 and 57% for those same areas, respectively. The table also includes the frequency of a household member's water-related diseases (FWRD) and cardiovascular diseases (FCVD) morbidities across varying arsenic contamination levels under salinity. FWRD morbidity ranges from one to four diseases (FWRD-1 to FWRD-4), while FCVD morbidity ranges from one to three (FCVD-1 to FCVD-3). The percentages of households suffering from FWRD-1, FWRD-2, FWRD-3 and FWRD-4 are 26, 5, 1 and 1% in no, 26, 11, 2 and 1% in medium, 37, 9, 2 and 1% in high arsenic areas under salinity, respectively. Similarly, the percentages of households suffering from FCVD-1, FCVD-2 and FCVD-3 are 14, 1 and 0% in no, 16, 5 and 1% in medium, 19, 2 and 0% in high arsenic areas under salinity, respectively. Figure 3 further illustrates the percentages of WRD, CVD and AD morbidities in arsenic areas, showing higher morbidity rates in medium and high arsenic areas than in no arsenic areas under salinity. Specifically, WRD (CVD) morbidity rates are 27% (25%) in no, rising to 33% (39%) in medium and 40% (36%) in high arsenic areas under salinity. AD morbidity rates follow a similar pattern, with 28, 33 and 39% in no, medium and high arsenic areas under salinity, respectively. The statistics indicate an upward trend in disease morbidity linked with increasing arsenic contamination under salinity in the study areas.

**Table 2:** Summary statistics of the dependent variables by areas.

**Variables**	**Arsenic areas under salinity**			**Overall**
	**No**	**Medium**	**High**	
**Water-related diseases (WRD)**				
Average (median)^a^	0.34 (0.00)	0.39 (0.00)	0.48 (0.00)	0.40 (0.00)
SD^b^	0.47	0.49	0.50	0.49
**Cardiovascular diseases (CVD)**				
Average (median)	0.16 (0.00)	0.22 (0.00)	0.21 (0.00)	0.20 (0.00)
SD	0.36	0.42	0.41	0.40
**Any diseases (AD)**				
Average (median)	0.41 (0.00)	0.48 (0.00)	0.57 (1.00)	0.48 (0.00)
SD	0.49	0.50	0.50	0.50
**Frequency of household with one/several water-related**				
**diseases (FWRD)**				
**FWRD-1**				
Average (median)	0.26 (0.00)	0.26 (0.00)	0.37 (0.00)	0.29 (0.00)
SD	0.44	0.44	0.48	0.45
**FWRD-2**				
Average (median)	0.05 (0.00)	0.11 (0.00)	0.09 (0.00)	0.08 (0.00)
SD	0.23	0.31	0.29	0.28
**FWRD-3**				
Average (median)	0.01 (0.00)	0.02 (0.00)	0.02 (0.00)	0.02(0.00)
SD	0.11	0.13	0.12	0.12
**FWRD-4**				
Average (median)	0.01 (0.00)	0.01 (0.00)	0.01 (0.00)	0.01(0.00)
SD	0.07	0.09	0.07	0.08
**Frequency of household with one/several cardiovascular**				
**diseases (FCVD)**				
**FCVD-1**				
Average (median)	0.14 (0.00)	0.16 (0.00)	0.19 (0.00)	0.16 (0.00)
SD	0.35	0.37	0.39	0.37
**FCVD-2**				
Average (median)	0.01(0.00)	0.05 (0.00)	0.02 (0.00)	0.03 (0.00)
SD	0.11	0.21	0.14	0.16
**FCVD-3**				
Average (median)	0.00 (0.00)	0.01 (0.00)	0.00 (0.00)	0.01 (0.00)
SD	0.00	0.11	0.00	0.07

FWRD-1 to FWRD-4 = the frequency of a household member suffered from 1 to 4 WRDs simultaneously.

FCVD-1 to FCVD-3 = the frequency of a household member suffered from 1 to 3 CVDs simultaneously.

The frequency of a household member suffered from FWRD-5 and FCVD-4 is negligible; therefore, the results are not included in the table.

^a^Median in parenthesis.

^b^SD, standard deviation.

Table 3 summarizes the average, median and standard deviation of independent variables, categorized into water uses, water safety measures and sociodemographic variables across different arsenic areas under salinity. The average drinking water (cooking water) use is 15.49 (18.77), 16.52 (15.47) and 18.86 (19.20) liters in no, medium and high arsenic areas under salinity, respectively. Regarding washing water, 13, 18 and 37% of households use < 100 L daily in no, medium and high arsenic areas under salinity, respectively. The use of deep tubewells (other) as a water safety measures is documented at 3% (55%) in no, 58% (38%) in medium and 4% (65%) in high arsenic areas under salinity. The average age across the overall sample is consistent at around 39 years. The percentage of females in the study areas is similar to that of males, with an average of 53% for females. Agriculture is the primary occupation for 13, 14 and 33% of households in no, medium and high arsenic areas under salinity, respectively. The average education level is 6–7 years of schooling across all areas. The dominant family structure is an extended family in the study areas, which is consistent in all areas, with about 75% prevalence. The average monthly income of the sample households is ~11,000 BDT, ranging from 9,000 to 13,000 BDT.

**Table 3:** Summary statistics of the independent variables by areas.

**Variables**	**Arsenic areas under salinity**			**Overall**
	**No**	**Medium**	**High**	
**Water uses**				
**Drinking (L)**				
Average (median)^a^	15.49 (15.00)	16.52 (15.00)	18.86 (15.00)	16.84 (15.00)
SD	19.71	7.81	9.89	13.66
**Cooking (L)**				
Average (median)	18.77 (15.00)	15.47 (15.00)	19.20 (20.00)	17.70 (15.00)
SD	13.35	8.03	11.89	11.35
**Washing (base group = more than 100 L)**				
Average (median)	0.13 (0.00)	0.18 (0.00)	0.37 (0.00)	0.22 (0.00)
SD	0.34	0.39	0.48	0.41
**Water safety measures (base group = rainwater)**				
**Deep tubewell**				
Average (median)	0.03 (0.00)	0.58 (1.00)	0.04 (0.00)	0.23 (0.00)
SD	0.16	0.49	0.20	0.42
**Other**				
Average (median)	0.55 (1.00)	0.38 (0.00)	0.65(1.00)	0.52 (1.00)
SD	0.50	0.49	0.48	0.50
**Sociodemographic variables**				
**Age**				
Average (median)	38.39 (35.00)	38.41 (35.00)	40.65 (40.00)	39.05 (36.00)
SD	14.16	15.28	15.00	14.83
**Gender (base group = male)**				
Average(median)	0.52 (1.00)	0.52 (1.00)	0.57 (1.00)	0.53 (1.00)
SD	0.50	0.50	0.50	0.50
**Occupation of HH (base group = non-agriculture)**				
Average (median)	0.13 (0.00)	0.14 (0.00)	0.33 (0.00)	0.19 (0.00)
SD	0.33	0.34	0.47	0.39
**Education of HH**				
Average (median)	5.98 (6.00)	6.35 (7.00)	7.33 (8.00)	6.50 (8.00)
SD	4.23	3.90	4.01	4.08
**Family structure (base group = nuclear)**				
Average (median)	0.75 (1.00)	0.76 (1.00)	0.72 (1.00)	0.75 (1.00)
SD	0.43	0.43	0.45	0.44
**Household income (in BDT)**				
Average (median)	9,410 (8,000)	11,433 (10,000)	13,067 (10,000)	11,205 (9,000)
SD	6,133.26	6,854.36	10,410.09	7,969.15

^a^Median in parenthesis.

SD, standard deviation; HH, household head; BDT, Bangladesh Taka.

Table 4 presents the marginal effects of the independent variables on WRD, CVD and AD morbidities in logit regression models 1, 2, and 3, respectively. High arsenic areas under salinity (all models), cooking water (model 2), washing water (models 1 and 3), and water safety measures (models 1 and 3) are significant at the 1 to 10% levels. The table shows that residing in high arsenic areas increases the probability of WRD, CVD, and AD morbidities by 15, 8 and 18%, respectively, compared to no arsenic areas under salinity. These results highlight the elevated health risks faced by residents of arsenic-contaminated coastal areas, which is consistent with prior studies. The literature demonstrates a positive associations between arsenic exposure and WRD morbidity (19, 20, 63, 64) and CVD morbidity (13, 65, 66). Furthermore, cooking water raises the likelihood of CVD morbidity by 0.3% per liter. Using < 100 L of washing water daily increases WRD and AD morbidities by 9 and 8%, respectively, compared to using >100 L. These elevated risks likely arise from the use of unsafe water for cooking and washing purposes. Deep tubewells (other) water increases WRD and AD morbidities by 17 (10) and 19% (9%), respectively, compared to rainwater, supporting the effectiveness of treated rainwater against the morbidity (12, 67). Overall, the results show that high arsenic contaminated areas increase the likelihood of morbidity, cooking (washing) water is linked to high CVD (WRD) morbidity, and rainwater is safer than deep tubewell water for reducing the health risks.

**Table 4:** Marginal effects of the independent variables on water-related diseases (WRD), cardiovascular diseases (CVD) and any diseases (AD) in logit regression

**Independent variables**	**Logit regression**		
	**WRD**	**CVD**	**AD**
	**Model 1**	**Model 2**	**Model 3**
**Arsenic areas under salinity (base group = no arsenic)**			
Medium	−0.02	0.06	−0.002
High	0.15***	0.08*	0.18***
**Water uses**			
Drinking	−0.001	−0.001	−0.001
Cooking	−0.001	0.003**	0.003
Washing (base group = more than 100 L)	0.09*	0.05	0.08*
**Water safety measures (base group = rainwater)**			
Deep tubewell	0.17**	0.06	0.19**
Other	0.10**	0.01	0.09*
**Sociodemographic variables**			
Age	0.004***	0.004***	0.01***
Gender (base group = male)	0.10**	0.03	0.10**
Occupation of HH (base group = non-agri.)	0.002	−0.05	−0.02
Education of HH	−0.002	−0.002	−0.001
Family structure (base group = nuclear)	0.09*	−0.02	0.06
Household income^a^	−0.01	−0.05*	−0.03
LR χ^2^	35.02	35.61	49.05
Number of observations	689		

WRD, water-related diseases; CVD, cardiovascular diseases; AD, any disease; HH, household head.

^a^ Regressions are performed using the natural logarithm of household income.

***Significant at 1 percent level, **at 5 percent level and *at 10 percent level.

For the robustness check, we introduced various models and same qualitative results are consistently observed across all models.

In Table 4, sociodemographic variables, such as age (all models), gender (models 1 and 3), family structure (model 1) and household income (model 2) are significant at 1 to 10% levels. Age is associated with 0.4, 0.4 and 1% increases in the probabilities of WRD, CVD and AD morbidities, respectively, for each additional year. This result aligns with the literature indicating elevated WRD (68, 69) and CVD (70, 71) morbidity among old individuals. Females are 10% more likely to experience WRD and AD morbidities than males, reflecting their extensive involvement in household water-related chores. These findings are consistent with research identifying greater female vulnerability to WRD morbidity than male vulnerability (72–74). Extended families are 9% more likely than nuclear families to face WRD morbidity. Finally, a 1% increase in household income reduces the probability of CVD morbidity by 5%, which aligns with studies that link high income to improved health outcomes (75, 76). Overall, old individuals, females, extended families and low household income increase the likelihood of WRD, CVD and AD morbidities.

Table 5 presents the marginal effects of the independent variables on the intensity or frequency of water-related diseases (FWRD) and cardiovascular diseases (FCVD) morbidities in ordered probit regression models 1 and 2, respectively. Model 1 includes the frequency of one, two or three WRD (FWRD-1, FWRD-2 or FWRD-3), whereas model 2 includes the frequency of one or two CVD (FCVD-1 or FCVD-2) morbidities simultaneously by a household member. The table indicates that high arsenic areas (models 1 and 2) and medium arsenic areas under salinity (model 2), cooking water (model 2), washing water (model 1) and water safety measures (model 1) are significant at 1 to 10% levels. Specifically, living in high arsenic areas elevates the likelihood of FWRD-1, FWRD-2 and FWRD-3 morbidity by ~7, 4 and 1%, respectively, compared to no arsenic areas under salinity. Similarly, medium (high) arsenic areas under salinity increase the probability of FCVD-1 and FCVD-2 morbidity by ~7 (6) and 1.6% (1%), respectively. These patterns indicate that living in arsenic contaminated areas, whether medium or high, increases the probability of both the incidence and intensity of WRD and CVD morbidities. Furthermore, each additional liter of cooking water increases the likelihood of FCVD-1 and FCVD-2 morbidity by 0.3 and 0.1%, respectively. Using < 100 L of water daily for washing raises the probability of FWRD-1, FWRD-2 and FWRD-3 morbidity by about 5, 3 and 1%, respectively, compared to using over 100 L. These findings underscore that depending on contaminated water sources for cooking and washing may contribute to the high intensity of the morbidities. Households relying on deep tubewells (other) water face a high probability of FWRD-1, FWRD-2 and FWRD-3 morbidity by around 10 (7), 6 (4) and 1% (1%), respectively, compared to rainwater. Table 6 presents the marginal effects of arsenic contamination, water uses and safety measures on the frequency of one to four any diseases (FAD-1, FAD-2, FAD-3 or FAD-4) morbidity simultaneously by a household member and reveals results consistent with those shown in Table 5 for FWRD and FCVD. Overall, the findings show that living in high arsenic contaminated areas increases the likelihood of morbidity frequency, cooking (washing) water is linked to a high CVD (WRD) morbidity frequency and relying on deep tubewells elevates morbidity risks compared to rainwater.

**Table 5:** Marginal effects of the independent variables on frequency of water-related diseases (FWRD) and cardiovascular diseases (FCVD) in ordered probit regression.

**Independent variables**	**FWRD**			**FCVD**	
	**FWRD-1**	**FWRD-2**	**FWRD-3**	**FCVD-1**	**FCVD-2**
	**Model 1**			**Model 2**	
**Arsenic areas under salinity (base group = no arsenic)**					
Medium	0.002	0.001	0.001	0.07*	0.016*
High	0.07***	0.04***	0.01**	0.06*	0.01*
**Water uses**					
Drinking	0.001	0.001	0.001	−0.001	−0.001
Cooking	0.001	0.001	0.001	0.003***	0.001**
Washing (base group = more than 100 L)	0.05**	0.03**	0.01*	0.04	0.01
**Water safety measures (base group = rainwater)**					
Deep tubewell	0.10**	0.06**	0.01**	0.04	0.01
Other	0.07**	0.04**	0.01**	0.003	0.001
**Sociodemographic variables**					
Age	0.002***	0.001***	0.001**	0.004***	0.001***
Gender (base group = male)	0.04**	0.03**	0.01*	0.02	0.01
Occupation of HH (base group = non-agri.)	−0.002	−0.001	−0.001	−0.03	−0.01
Education of HH	−0.001	−0.001	−0.001	−0.002	−0.001
Family structure (base group = nuclear)	0.03	0.02	0.004	−0.01	−0.002
Household income^a^	−0.01	−0.01	−0.002	−0.04*	−0.01*
LR χ^2^	35.41			43.74	
Number of observations	689				

HH, household head.

^a^Regressions are performed using the natural logarithm of household income.

***Significant at 1 percent level, **at 5 percent level and *at 10 percent level.

The frequency of a household member suffered from FWRD-4, FWRD-5 and FCVD-3, FCVD-4 is negligible; therefore, the results are not included in the table.

**Table 6:** Marginal effects of the independent variables on frequency of any diseases (FAD) in ordered probit regression.

**Independent variables**	**Frequency of any diseases (FAD)**			
	**FAD-1**	**FAD-2**	**FAD-3**	**FAD-4**
**Arsenic areas under salinity (base group = no arsenic)**				
Medium	0.02	0.01	0.01	0.01
High	0.06***	0.05***	0.02***	0.02***
**Water uses**				
Drinking	−0.001	−0.001	−0.001	−0.001
Cooking	0.001	0.001	0.001	0.001
Washing (base group = more than 100 L)	0.04**	0.03**	0.01*	0.01*
**Water safety measures (base group = rainwater)**				
Deep tubewell	0.07**	0.05**	0.03**	0.02**
Other	0.04**	0.03**	0.01*	0.01*
**Sociodemographic variables**				
Age	0.002***	0.002***	0.001***	0.001***
Gender (base group = male)	0.03**	0.03**	0.01**	0.01**
Occupation of HH (base group = non-agri.)	−0.01	−0.01	−0.004	−0.003
Education of HH	−0.001	−0.001	−0.001	−0.001
Family structure (base group = nuclear)	0.01	0.01	0.01	0.004
Household income^a^	−0.02	−0.02	−0.01	−0.01
LR χ^2^	53.10			
Number of observations	689			

HH, household head.

^a^Regressions are performed using the natural logarithm of household income.

***Significant at 1 percent level, **at 5 percent level and *at 10 percent level.

The frequency of a household member suffered from FAD-5, FAD-6 and FAD-7 is negligible; therefore, the results are not included in the table.

In Table 5, sociodemographic variables, such as age (models 1 and 2), gender (model 1) and household income (model 2) are significant at 1 to 10% levels. The table shows that each additional year of age is associated with an increase in the likelihood of FWRD-1, FWRD-2 and FWRD-3 (FCVD-1 and FCVD-2) morbidity by 0.2, 0.1 and 0.1% (0.4 and 0.1%), respectively. Females are about 4, 3 and 1% more likely to experience FWRD-1, FWRD-2 and FWRD-3 morbidity, respectively, than males. Finally, a 1% increase in household income decreases the likelihood of FCVD-1 and FCVD-2 morbidity by 4 and 1%, respectively. Table 6 also presents the marginal effects of sociodemographic variables on the frequency of one to four any diseases (FAD-1, FAD-2, FAD-3, or FAD-4) morbidity simultaneously by a household member, revealing results consistent with those shown in Table 5 for FWRD and FCVD. These results underscore the role of sociodemographic variables, such as age, gender and income, which significantly affect both the incidence and intensity of WRD and CVD morbidities.

The results from Figure 3 and Tables 4–6 highlight the influence of arsenic contamination under salinity, water uses and safety measures on WRD, CVD and AD morbidity rates in coastal Bangladesh. Figure 3 shows higher morbidity rates in medium to high arsenic areas than in no arsenic areas under salinity. The logit regression models in Table 4 reveal that residing in high arsenic areas significantly increases the likelihood of WRD, CVD and AD morbidities compared to no arsenic areas under salinity. Ordered probit models in Tables 5, 6 further indicate greater intensity or frequency of WRD, CVD and AD morbidities in high arsenic areas than in no arsenic areas under salinity. The models also emphasize the impact of household water use patterns on health outcomes. Specifically, increased cooking water use raises CVD morbidity, while using < 100 L of washing water daily elevates WRD morbidity. In contrast, drinking water use has no significant effect on the health risks. Additionally, the use of deep tubewells water as a safety measure increases morbidity rates compared to the use of rainwater. These results address the research question of how morbidities from WRD and CVD are associated with arsenic contamination under salinity across multiple water sources and uses and how such risks can be reduced. We hypothesize that WRD and/or CVD morbidity rates worsen when severe arsenic contamination comes with salinity, and there exist effective countermeasures for the risk reduction by different channels. The results reveal that contamination elevates morbidity primarily through cooking and washing water channels, and deep tubewells appear less protective than rainwater. Overall, the analyses confirm that arsenic contamination under salinity through various channels increases the health risks and propose countermeasures to reduce these risks.

## 5 Discussion

In arsenic and salinity contaminated coastal Bangladesh, collaborative efforts by the government, non-governmental organizations (NGOs) and researchers have widely promoted interventions such as rainwater harvesting and deep tubewell installation to ensure safe drinking water (30, 32, 77–80). Our analysis revealed no significant link between drinking water use and morbidity, indicating that these interventions may have effectively reduced drinking-water–related health risks (7, 81). However, the water used for cooking and washing still poses significant health risks. The use of contaminated cooking water is associated with CVD morbidity, whereas the use of washing water is linked to elevated rates of WRD. Households lacking sufficient access to rainwater and deep tubewells typically rely on shallow tubewells and ponds for daily cooking and washing activities (82, 83). Ensuring the safety of these various water sources can be challenging because of logistical constraints, costs and the underestimation of associated risks (84, 85). Consequently, these often-overlooked channels, particularly those related to cooking and washing, can pose significant health risks to coastal inhabitants.

We also find that households relying on deep tubewells as a safety measure experience higher WRD morbidity than those using rainwater. This outcome is likely due to the overextraction of deep groundwater, which can lead to the downward migration of contaminated shallow water into deep aquifers (86, 87). Although deep tubewells are often considered a reliable option, their effectiveness may be declining in contaminated areas with significant pressure on groundwater resources. These situations highlight the necessity of broadening water safety measures beyond just drinking water. Practical steps could include, first, promoting year-round use of treated rainwater for cooking, washing and drinking by expanding household storage tank capacity and constructing community-level reservoirs provided at subsidized cost by the government and NGOs. Second, conducting routine monitoring of all water sources through community-NGO partnerships to enable regular testing of drinking, cooking and washing water at minimal cost. These integrated actions, along with awareness campaigns, are crucial for alleviating the disease risks in coastal areas and supporting the Sustainable Development Goal 3.

## 6 Conclusion

This study examines how morbidity from water-related diseases (WRD) and cardiovascular diseases (CVD) is associated with arsenic contamination under salinity and how such risks can be reduced. The results reveal that, first, households in high arsenic contaminated areas have higher morbidity rates of WRD and/or CVD than those in no arsenic contaminated areas under salinity. Second, a daily use of washing and cooking water (drinking water) increases (does not increase) the probability of WRD and CVD morbidities, respectively. Third, households using deep tubewells as a safety measure face greater WRD morbidity risks than those using rainwater. The results suggest that cooking and washing are the main channels for increasing the health risks, and two countermeasures are recommended: (i) extensive year-round uses of rainwater and (ii) adoption of regularly tested water sources, such as groundwater, even for cooking and washing in addition to drinking, to reduce risks. The study highlights the importance of integrated policies to manage different water uses and sources to achieve Sustainable Development Goal 3. The novel aspects of this study are (i) it analyzes the impact of the interaction between arsenic contamination and salinity on WRD and CVD morbidities rather than examining these exposures independently, (ii) it assesses health risks associated with different household water-use channels, such as drinking, cooking and washing through logit and ordered probit models to quantify the incidence and intensity of the morbidities and (iii) it offers evidence-based recommendations for affected coastal areas, which include providing subsidized rainwater storage facilities and fostering partnerships between communities and non-governmental organizations for the regular testing of all water sources.

We acknowledge that this study has several limitations, focusing on unmeasured confounders, dietary factors, seasonal data and biomarker validation. For each limitation, we propose specific directions for future research. First, we must admit that there may be additional determinants of WRD and CVD morbidities, such as household hygiene practices and food quality, that are not included in the statistical analyses. Future studies should incorporate such behavioral and nutritional factors to obtain a comprehensive understanding of health outcomes. Second, we did not account for individual dietary habits, such as salt and fat intake, which are relevant to CVD (88–90). Future research could integrate detailed dietary surveys to isolate the effects of arsenic and salinity from those of other dietary contributors. Third, the study is based on cross-sectional data collected during the dry season, when arsenic and salinity contamination reach peak levels (51, 91, 92). To capture seasonal fluctuations in contamination levels and health impacts, future studies should collect longitudinal or multiseasonal data. Finally, our study relied on self-reported health outcomes, and water uses and sources data rather than biological test data (e.g., blood and urine arsenic levels). This may introduce measurement bias. In future research, interdisciplinary collaboration with medical scientists could enable the integration of environmental surveys with biological testing to validate exposure pathways and strengthen causal inference. These caveats notwithstanding, we believe that this study is an important initial step in clarifying the relationship between arsenic contamination under salinity and the health risks, as inhabitants in coastal areas have been increasingly threatened by climate change and environmental degradation over time.

## Data Availability

The raw data supporting the conclusions of this article will be made available by the authors, without undue reservation.

## Footnotes

^1^The existing secondary data on arsenic levels is over a decade old, which led us to conduct limited field tests to verify its relevance. The limited tests confirm that the measured values are aligned with the previous data.

^2^Due to the scarcity of safe water, household members are cautious about its use, particularly for drinking and cooking, and are typically able to report their daily consumption with reasonable accuracy. However, for washing purposes, respondents generally provided only an approximate estimate, commonly indicating whether they use more or less than 100 L/day.

## Appendix

**APPENDIX A1:** Water sources and arsenic concentrations in drinking, cooking and washing water from 10 randomly selected households in each subdistrict.

**ID**	**Drinking water**		**Cooking water**		**Washing water**	
	**Source**	**Arsenic (ppb)**	**Source**	**Arsenic (ppb)**	**Source**	**Arsenic (ppb)**
**Shyamnagar**						
Household_01	5	0	4	0	1	10
Household_02	1	10	4	10	1	10
Household_03	5	0	4	10	4	10
Household_04	3	0	1	10	1	10
Household_05	1	10	1	10	1	10
Household_06	3	10	3	10	4	0
Household_07	5	0	3	10	4	0
Household_08	5	0	4	10	4	10
Household_09	5	0	1	10	1	10
Household_10	5	0	4	10	4	10
Average		3		9		8
**Debhata**						
Household_01	2	10	2	10	4	0
Household_02	2	0	2	0	1	100
Household_03	2	0	1	100	1	100
Household_04	2	50	2	50	4	0
Household_05	2	10	2	10	1	100
Household_06	2	10	2	10	4	0
Household_07	2	10	2	10	4	0
Household_08	1	50	1	50	1	50
Household_09	2	50	2	50	1	100
Household_10	2	0	2	0	1	50
Average		19		29		50
**Koyra**						
Household_01	1	100	1	100	1	100
Household_02	1	50	1	50	1	50
Household_03	5	0	1	25	1	25
Household_04	2	100	1	100	1	100
Household_05	5	0	1	100	1	100
Household_06	5	0	1	25	1	25
Household_07	3	0	1	100	1	100
Household_08	4	10	4	10	1	10
Household_09	3	0	4	0	1	250
Household_10	5	0	4	0	1	10
Average		26		51		77

Water sources: 1 = tubewell, 2 = deep tubewell, 3 = supply water, 4 = ponds, 5 = rain.

ppb, parts per billion.
